# Kaposi sarcoma incidence and mortality trends and disparities in the United States

**DOI:** 10.1186/s13027-025-00710-x

**Published:** 2025-11-04

**Authors:** Ahsan Raza Raja, Philippos Apolinario Costa, Muhammad Hyder Junejo

**Affiliations:** 1 Department of Medicine, Aga Khan University, Karachi, Sindh Pakistan; 2 Department of Medicine, Division of Oncology, Yale School of Medicine, New Haven, CT USA; 3 Department of Dermatology, Yale School of Medicine, New Haven, CT USA

**Keywords:** Kaposi sarcoma, Incidence, Mortality, Disparities, Epidemiology, HIV

## Abstract

**Background:**

Kaposi sarcoma (KS) is an angioproliferative tumor caused by human herpesvirus 8 and is an AIDS-defining illness. Combination antiretroviral therapy (ART) was introduced in the United States in 1996, after which U.S. KS incidence declined and survival improved; however, persistent racial and sex disparities remain. Our aim was to characterize 1999–2020 trends in KS incidence and mortality in the United States overall, and by race and sex, and to quantify changes in disparities over time.

**Methods:**

We extracted KS case and death counts from the CDC WONDER database (ICD-10 codes B21.0, C46). Age-adjusted incidence rates (AAIR) and mortality rates (AAMR) per 100,000 were calculated by year, race, and sex. Temporal trends were evaluated using the Mann–Kendall test (Kendall’s τ), and between-group differences by t-test (α = 0.05).

**Results:**

From 1999 to 2020, 27,886 KS cases and 4,380 deaths occurred. Overall AAIR was 0.99 in men versus 0.10 in women, and AAMR 0.16 versus 0.01 (both *p* < 0.001). Black men experienced the highest AAIR (2.23) and AAMR (0.40), significantly exceeding White men (0.79 and 0.13; *p* < 0.001). Incidence declined in both sexes (men: -46.7%, τ = -0.920; women: -58.9%, τ = -0.848; both *p* < 0.001). Mortality declined in men (-66.4%, τ = -0.581; *p* < 0.001). Among women, AAMR levels were very low throughout (mean ≈ 0.01 per 100,000); the end-to-end change from 1999 to 2020 was + 28.6%, yet the Mann–Kendall test did not identify a monotonic trend (τ = − 0.303; *p* = 0.060), reflecting early declines followed by year-to-year fluctuation around a low baseline.

**Conclusion:**

Although KS incidence and mortality have declined markedly since 1999, Black men remain disproportionately affected. Focused public health efforts and enhanced access to HIV care are essential to close these gaps.

**Clinical trial number:**

Not applicable.

## Introduction

Kaposi sarcoma (KS) is an angioproliferative tumor caused by human herpesvirus 8, which emerged prominently as an AIDS-defining illness in the early 1980s [1]. Combination antiretroviral therapy (ART) was introduced in the United States in 1996, after which overall U.S. KS incidence declined and survival improved [2–4]. However, KS remains a clinical concern: risk in people with HIV is still many-fold higher than in the general population [5]. Epidemiologic studies from 2000 to 2013 show that Black individuals experienced smaller declines in KS incidence and continue to bear a disproportionate burden compared with White individuals [4, 6]. KS predominantly affects men, and overall incidence increases with age [4, 7].

Our study assesses incidence and mortality trends over a 22-year period (1999–2020) using CDC WONDER data. To our knowledge, no prior analysis has applied rigorous statistical testing to quantify recent changes in sex- and race-specific disparities in both KS incidence and mortality. Identifying these demographic gaps is critical for dermatologists and public health practitioners to target interventions effectively and reduce ongoing inequities.

## Methods

We analyzed KS-associated incidence and mortality data for males and females in the United States from 1999 to 2020 using the Centers for Disease Control and Prevention’s Wide-Ranging Online Data for Epidemiologic Research (CDC WONDER) database, identifying cases by ICD-10 codes B21.0 and C46 [8]. This study utilized publicly available, de-identified data and was exempt from institutional review board approval. To maintain confidentiality, the database labels case counts fewer than ten as “Suppressed” and counts between ten and nineteen as “Unreliable” (Table 1). Consequently, we excluded individuals aged ≤ 14 years. Age-adjusted incidence rates (AAIR) and Age-adjusted mortality rates (AAMR) per 100,000 people were calculated by year, race, sex, and age group. The Mann-Kendall trend test was employed to assess incidence and mortality rate trends via the Kendall tau coefficient. Continuous variables were analyzed using t-tests to compare group differences. Moreover, annual incidence and mortality data for Asian or Pacific Islander (AAPI) individuals, American Indian or Alaska Native (AIAN) individuals, and for Black males aged ≥ 65 years, as well as mortality data for female subgroups stratified by age and race were either suppressed or deemed unreliable, t-test comparisons for these categories could not be calculated, and only pooled overall rates are presented in Table 1. All statistical analyses were performed in RStudio, with statistical significance set at *p* < 0.05.

**Table 1:** Mean age-adjusted rates of Kaposi sarcoma incidence and mortality stratified by sex and age group

	Female (95% CI)	Male (95% CI)		Age 15–64 years (95% CI)	Age ≥ 65 years (95% CI)
Age-adjusted incidence rate per 100,000					
All	0.10 (0.10–0.11)	0.99 (0.98–1.00)	White men	0.70 (0.69–0.72)	1.26 (1.22–1.30)
White	0.08 (0.07–0.08)	0.79 (0.78–0.81)	Black men	2.39 (2.33–2.45)	1.41 (1.28–1.56)
Black	0.24 (0.22–0.26)	2.23 (2.18–2.29)	White women	0.02 (0.02–0.03)	0.36 (0.35–0.38)
Asian or Pacific Islander	0.04 (0.03–0.05)	0.42 (0.39–0.46)	Black women	0.22 (0.20–0.23)	0.36 (0.31–0.42)
American Indian or Alaska Native	Suppressed	0.55 (0.46–0.65)	Asian or Pacific Islander men	0.34 (0.31–0.37)	0.85 (0.70–1.02)
Age 15–64 years	0.05 (0.05–0.05)	0.93 (0.92–0.94)	Asian or Pacific Islander women	0.01 (0.01–0.02)	0.19 (0.13–0.26)
Age ≥ 65 years	0.37 (0.35–0.39)	1.30 (1.26–1.33)	American Indian or Alaska Native men	0.53 (0.44–0.62)	0.68 (0.38–1.15)
			American Indian or Alaska Native women	Suppressed	Suppressed
Age-adjusted mortality rate per 100,000					
All	0.01 (0.01–0.01)	0.16 (0.16–0.17)	White men	0.10 (0.10–0.11)	0.26 (0.24–0.28)
White	0.01 (0.01–0.02)	0.13 (0.12–0.13)	Black men	0.43 (0.40–0.45)	0.28 (0.23–0.35)
Black	0.03 (0.02–0.04)	0.40 (0.38–0.43)	White women	0.00 (0.00–0.00)	0.08 (0.08–0.09)
Asian or Pacific Islander	Unreliable	0.05 (0.04–0.07)	Black women	0.02 (0.02–0.03)	0.06 (0.04–0.09)
American Indian or Alaska Native	Suppressed	0.10 (0.06–0.16)	Asian or Pacific Islander men	0.03 (0.02–0.05)	0.16 (0.10–0.25)
Age 15–64 years	0.00 (0.00–0.00)	0.14 (0.14–0.15)	Asian or Pacific Islander women	Suppressed	Suppressed
Age ≥ 65 years	0.08 (0.08–0.09)	0.25 (0.23–0.26)	American Indian or Alaska Native men	Unreliable	Suppressed
			American Indian or Alaska Native women	Suppressed	Suppressed

*Suppressed: count < 10; Unreliable: count 10–19 per CDC WONDER definitions

## Results

Between 1999–2020, 27,886 new KS cases and 4,380 KS deaths were reported in the U.S. The male-to-female incidence ratio was 8:1 and mortality 6:1. KS cases were 18,370 (65.9%) White, 7,851 (28.2%) Black, 591 (2.1% ) AAPI, 174 (0.6%) AIAN, and 900 (3.2%) other race; deaths were 2,870 (65.5%) White, 1,399 (31.9%) Black, 82 (1.9%) AAPI, 29 (0.7%) AIAN, and no deaths were recorded in the ‘other race’ category. The overall AAIR and AAMR were 0.53 (95% CI 0.53–0.54) and 0.07 (95% CI 0.07–0.08) per 100,000 persons, respectively.

During 1999–2020, the AAIR of KS was significantly higher in men than women. Overall AAIR was 0.99 per 100,000 (95% CI 0.98–1.00) in men versus 0.10 (0.10–0.11) in women (*p* < 0.001) (Table 1). By race, Black individuals had the highest incidence: AAIR was 2.23 (2.18–2.29) in Black men and 0.24 (0.22–0.26) in Black women, significantly exceeding rates in White persons (0.79 [0.78–0.81] in White men; 0.08 [0.07–0.08] in White women; *p* < 0.001 for all comparisons). AAPI had the lowest AAIR (0.42 [0.39–0.46] in men; 0.04 [0.03–0.05] in women), and AAIR data for AIAN women were suppressed (AIAN men 0.55 [0.46–0.65]). Furthermore, AAIR was substantially higher in older adults: persons ≥ 65 years had AAIR of 1.30 (1.26–1.33) in men and 0.37 (0.35–0.39) in women, compared to 0.93 (0.92–0.94) and 0.05 (0.05–0.05) in ages 15–64 (*p* < 0.001 for each sex).

AAMR showed similar disparities. Overall AAMR was 0.16 (0.16–0.17) in men versus 0.01 (0.01–0.01) in women (*p* < 0.001) (Table 1). Among men, Black individuals again had the highest mortality (0.40 [0.38–0.43] per 100,000), versus 0.13 (0.12–0.13) in White men (*p* < 0.001). Black women had higher AAMR (0.03 [0.02–0.04]) than White women (0.01 [0.01–0.02]; *p*-value not calculable due to count “suppression” in females when stratified by race). AAPI men had AAMR of 0.05 (0.04–0.07) (AAPI women’s rate was classified as unreliable) and AIAN men 0.10 (0.06–0.16) (AIAN women suppressed). Mortality also increased with age: in men, AAMR was 0.25 (0.23–0.26) in ≥ 65 vs. 0.14 (0.14–0.15) in 15–64 (*p* < 0.001); in women, it was 0.08 (0.08–0.09) in ≥ 65 vs. 0.00 (0.00–0.00) in 15–64 (*p*-value not calculable due to count “suppression” in females when stratified by age).

**Fig. 1:**
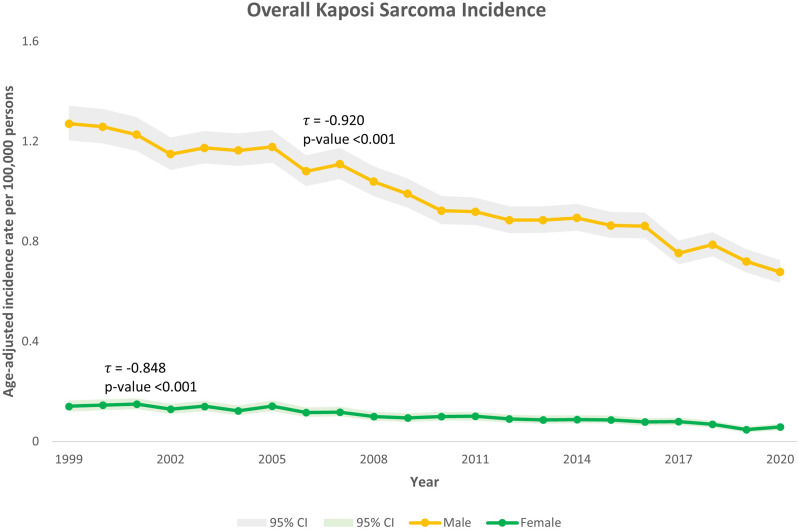
Trends in all Kaposi Sarcoma-related incidence in the United States, 1999–2020

**Fig. 2:**
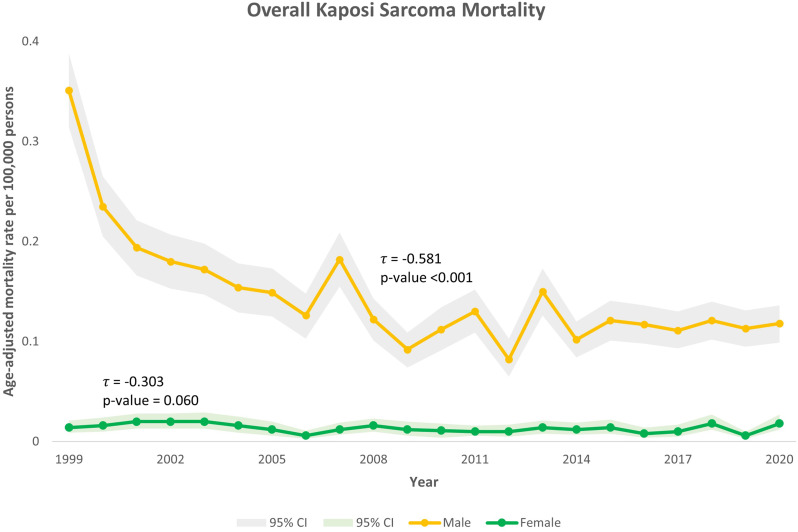
Trends in all Kaposi Sarcoma-related mortality in the United States, 1999–2020

Over 1999–2020, AAIR declined in both sexes: Kendall’s τ = -0.920 for men and − 0.848 for women (both *p* < 0.001; Fig. 1), corresponding to 46.7% and 58.9% decreases, respectively. By contrast, AAMR trends diverged by sex (Fig. 2). Men showed a significant decline (τ = -0.581; *p* < 0.001; -66.4%). Among women, AAMR levels were very low throughout (mean ≈ 0.01 per 100,000). Although the endpoint change from 1999 to 2020 was + 28.6%, the Mann–Kendall test did not identify a monotonic trend (τ = -0.303; *p* = 0.060), consistent with an early decrease followed by low-level year-to-year fluctuation.

**Fig. 3:**
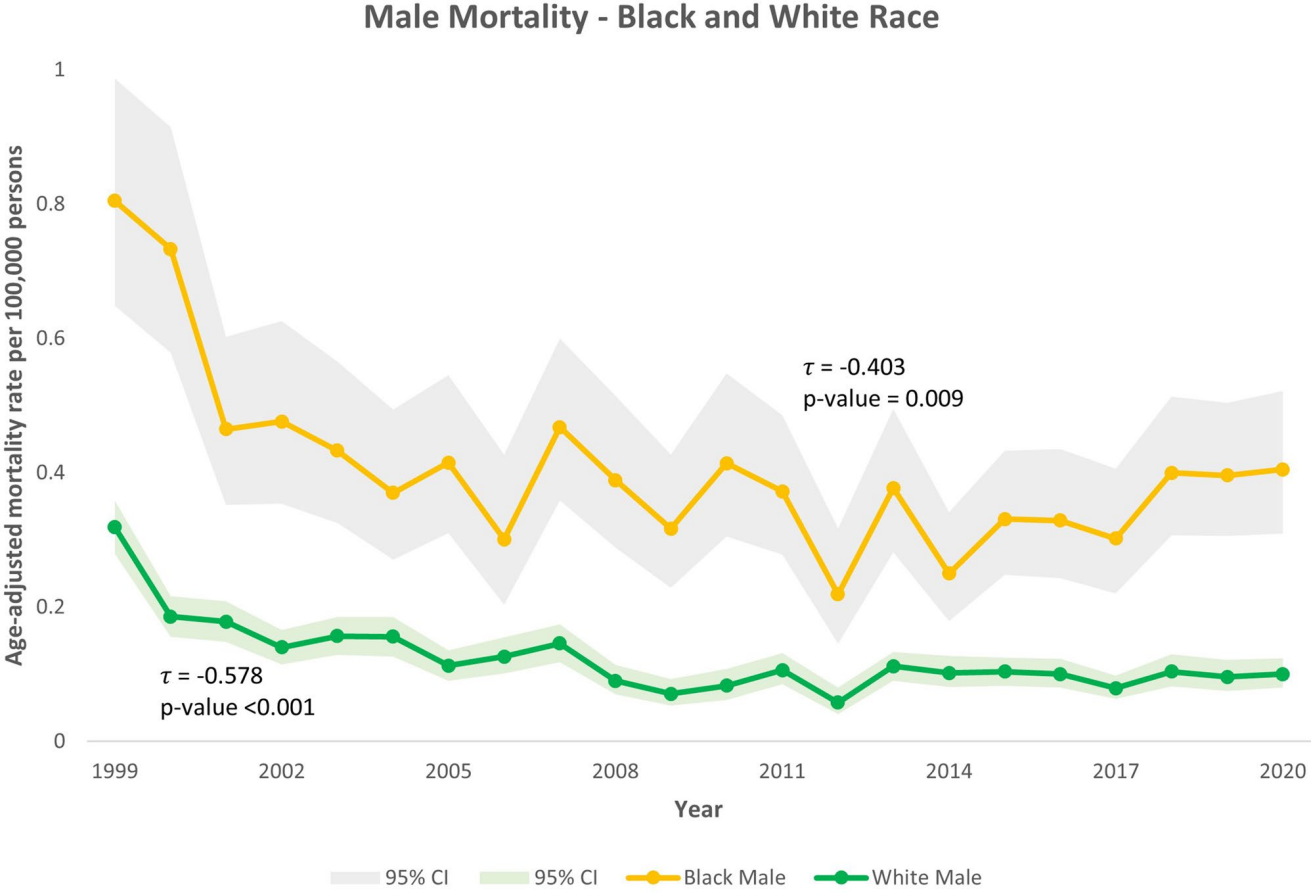
Trends in Kaposi Sarcoma-related mortality among males stratified by race in the United States, 1999–2020

**Fig. 4:**
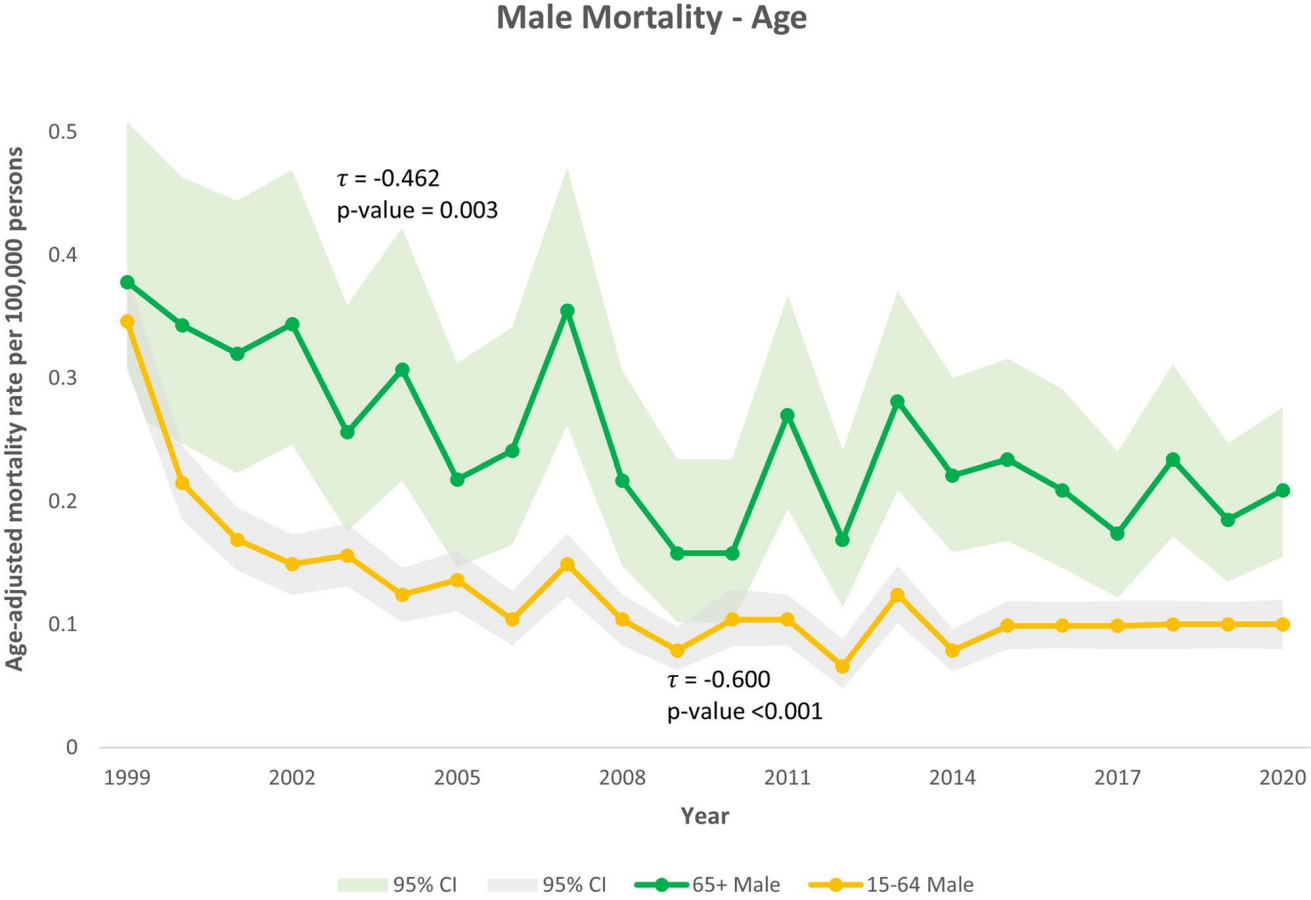
Trends in Kaposi Sarcoma-related mortality among males stratified by age of death in the United States, 1999–2020

Among male subgroups, mortality declined for both Black and White men. For White men, τ = -0.578 (*p* < 0.001), and for Black men τ = -0.403 (*p* = 0.009) (Fig. 3). By age in men, mortality also declined in both groups: τ = -0.600 for ages 15–64 (*p* < 0.001) and τ = -0.462 for ages ≥ 65 (*p* = 0.003) (Fig. 4).

## Discussion

Our analysis highlights two major findings in the U.S. KS landscape: (1) a marked decline in incidence and mortality rates since 1999, and (2) significant racial and sex disparities that disproportionately affect Black individuals, particularly Black men. These trends align with broader epidemiological shifts following the introduction of ART [1, 5], yet they also underscore systemic inequities in healthcare access and outcomes.

The decline in KS incidence and mortality, most pronounced among males, reflects the transformative impact of ART on HIV-associated KS. At the height of the AIDS epidemic, KS incidence reached 47 cases per million people, but widespread ART adoption reduced this to ~ 6 cases per million [9]. Our data corroborate these changes, showing a 46.7% decline in male incidence and a 66.4% decline in male mortality from 1999 to 2020.

Racial disparities remain pronounced. Black men had 182.3% higher incidence and 207.7% higher mortality than White men. Although KS is rare in women, Black women had a 200% higher incidence and mortality than White women. These findings align with Surveillance, Epidemiology, and End Results (SEER) data demonstrating higher incidence (IR: 1.140 vs. 0.486) and lower 5-year survival (57.38% vs. 74.79%) among Black vs. White individuals [9]. Notably, Black men aged 15–64 had the highest incidence (2.39 per 100,000) and mortality (0.43 per 100,000). A recent study similarly reported a 3.3% significant increase in incidence among young Black men specifically in the southern U.S [10].

These disparities likely arise from multiple factors. Black individuals tend to present with KS at a younger age and with more advanced disease at the time of diagnosis, though the underlying reasons behind this observation remain unclear [11]. Systemic disparities in healthcare for the Black population may be contributing factors, possibly leading to inadequate viral suppression, delayed care, and late ART initiation—resulting in more advanced KS at diagnosis [12, 13]. Previous studies have shown that advanced KS correlates with higher mortality [14]. The CDC’s HIV Continuum of Care Reports have highlighted that Black males experience the lowest levels of care and viral suppression [15].

Underrepresentation in clinical trials further limits access to cutting-edge therapies. Black individuals represent 28.2% of KS incidence but only 21.4% of trial participants [16]. Moreover, gaps in health insurance coverage present significant barriers to effective KS management. According to the 2016 National Health Interview Survey, 15% of Black adults aged 18 to 64 were uninsured at the time of the interview, compared to 8.6% of White adults [17], further complicating access to consistent care for KS in the Black population.

Additionally, persistent disparities among Black individuals may also be related to an increase in organ transplants within this demographic. According to the Organ Procurement and Transplantation Network (OPTN), transplant rates for Black individuals rose in 2015, while those for White individuals decreased [18]. Furthermore, the HIV Organ Policy Equity (HOPE) Act, signed into law in 2013, allowed for HIV-to-HIV organ transplants, which is particularly relevant for Black populations due to the prevalence of HIV associated nephropathy. In the US, the first HIV-to-HIV transplant was performed in 2016, and by September 2020, 223 such transplants had been performed [19]. Individuals who undergo organ transplantation are 125 times more likely to develop KS, and HIV patients are 451 times more likely to do so compared to the general population [20]. The dual immunosuppression resulting from HIV infection and transplant immunosuppressive therapy likely exacerbates KS-related mortality in this population, and formal studies in this are underway [21]. Migration from endemic KS regions, such as Sub-Saharan Africa, may also contribute to the persistently high KS mortality. As of 2019, approximately 2.1 million immigrants from Sub-Saharan Africa were living in the United States, a 16-fold increase from 1980 [22].

Using CDC WONDER confines our analysis to aggregate, de-identified data without patient-level information on HIV status, ART adherence, comorbidities, or socioeconomic status. “Suppressed” counts (< 10) and “unreliable” counts (10–19) prevented subgroup analyses for AAPI and AIAN individuals, Black men ≥ 65, and female strata by age and sex. Dependence on ICD-10 codes may under- or misclassify KS and reflect coding changes over time; CDC WONDER also does not distinguish among the five clinical KS subtypes (classic, endemic, epidemic, iatrogenic, and nonepidemic), limiting subtype-specific analyses. As an ecological time-trend study, we cannot establish causality or adjust for unmeasured confounders. Strengths of this work include its national, population-based scope over 22 years; standardized age-adjusted incidence and mortality estimates; granular stratification by sex, race, and age; and rigorous Mann–Kendall trend testing, all using a reproducible public dataset with transparent handling of small-cell suppression.

## Conclusion

Despite substantial progress in KS management, pronounced racial and sex disparities persist, paralleling broader inequities in HIV care and cancer outcomes across the United States. Addressing these gaps requires integrated strategies combining ART optimization, culturally competent care, and policies to dismantle structural barriers to health equity. Healthcare providers should remain vigilant in diagnosing and treating KS, particularly in Black men that are disproportionately affected.

## Data Availability

The datasets analyzed during the current study are available in the CDC WONDER repository (https://wonder.cdc.gov/cancer.html).

## Abbreviations

KS: Kaposi sarcoma
AIDS: Acquired immunodeficiency syndrome
ART: Combination antiretroviral therapy
U.S.: United States
HIV: Human immunodeficiency virus
AAIR: Age-adjusted incidence rate
AAMR: Age-adjusted mortality rate
CDC WONDER: Centers for Disease Control and Prevention Wide-Ranging Online Data for Epidemiologic Research
AAPI: Asian or Pacific Islander
AIAN: American Indian or Alaska Native
*p*: *p*-value
CI: Confidence interval
